# Congenital Insensitivity to Pain with Anhidrosis (CIPA): A Case Report

**Published:** 2011-02-01

**Authors:** A Safari, A A Khaledi, M Vojdani

**Affiliations:** 1 Department of Prosthodontic, Faculty of Dentistry, Shiraz University of Medical Sciences, Shiraz, Iran

**Keywords:** Congenital, Insensitivity, Pain, Anhidrosis, Iran

## Abstract

Congenital insensitivity to pain with anhidrosis (CIPA) is a rare disorder characterized by episodes of fever and the inability to sense of pain despite the fact that all other sensory modalities remain intact or minimally impaired. The patient also may exhibit the signs of self-mutilation, mental retardation and little or no perspiration. We present a 10 years old Iranian patient diagnosed with CIPA with the above-mentioned clinical characteristics. The prosthetic treatment and the subsequent six month follow-up are discussed. Follow-up of the patient revealed that, with the use of this prosthesis, the patient’s oral function and esthetics were established and the mouth lesions improved. Therefore especial dental management of CIPA patients according to their mental status, age, oral and dental condition is essential for solving the specific problems each case may present and the full mouth teeth extraction should be considered as the last treatment.

## Introduction

Congenital insensitivity to pain with anhidrosis (CIPA) or hereditary sensory; type IV autonomic neuropathy is an extremely rare autosomal recessive disorder.[1] Less than 60 cases are available in the medical literature.[2] CIPA is characterized by congenital pain insensitivity, lack of thermal sensitivity, mental retardation of varying intensities, self-mutilation, little or no perspiration, failure to thrive and recurrent episodes of fever secondary to anhidrosis. The height and weight for these patients are below normal for their age.[2][3][4][5][6][7][8]

Patients with CIPA show an absence of unmyelinated fibers in addition to the loss of small myelinated ones.[9][10] This results into absence of epidermal nerve fibers and fibers around the sweat glands form the morphological basis of analgesia and anhidrosis in CIPA. Sense of touch and pressure sensitivity are not impaired in these patients.[1] Self-mutilating behavior due to a lack of pain perception is frequently seen in these patients. CIPA patients may injure themselves using their teeth.[11] Finger biting, laceration and ulceration of the tongue; lips and other oral mucosa are frequently observed. Furthermore, dental luxation and severe attrition are normally found. Fibrous scars in the cheeks due to repeated bites can decrease the mouth’s ability to open. These ulcers lead to several local and systemic problems, such as infection, tongue bleeding, malnutrition, halitosis and chronic osteomyelitis. Oral self-mutilations are found to decrease with age and with social and/or emotional development of the patients, however they are not completely eliminated.[12][13]

This report aims to describe a case of 10 year-old girl with CIPA characteristics. Dental treatment of this rare disorder is discussed. The patient's response to this treatment was evaluated during a 6 months period.

## Case Report

A 10 year-old girl was referred to the Prosthodontic Department, School of Dentistry, Shiraz University of Medical Sciences, Shiraz, Iran by her parents who were in search of a prosthesis that could prevent injuries to her oral mucosa, and improve her function and appearance. Medical history revealed that the girl was the second child of consanguineous parents whose first child was normal. When the patient was a few months old, her parents began to suspect that there was something wrong with the child since she failed to cry during painful stimuli, did not sweat and suffered from episodes of unexplained fever that decreased with physical activity. By the time she was 12 months old, deep ulcers developed in the patient’s fingers, lips, tongue and oral mucosa due to bites, with premature loss of a number of teeth due to biting hard toys. At that time, the child was diagnosed with CIPA however, the parents did not seek dental management until she was 10 years old.

Oral examination of the child showed mixed dentition. The left and right permanent canines were present in the mandible; however other teeth were lost due to biting hard items and self-tooth extraction. The remaining mandibular teeth had mild cervical caries.

**Fig. 1A and 1B:**
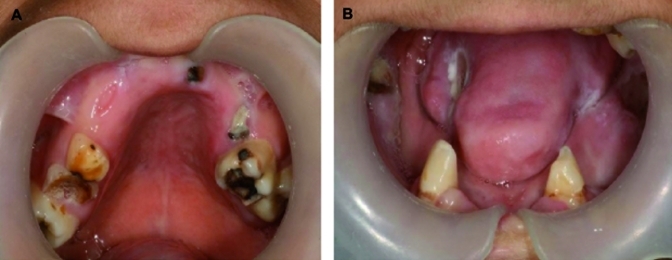
First clinical oral examination of the patient: Mandibular and maxillary remained teeth and mandibular mutilated mucosa

**Fig. 2:**
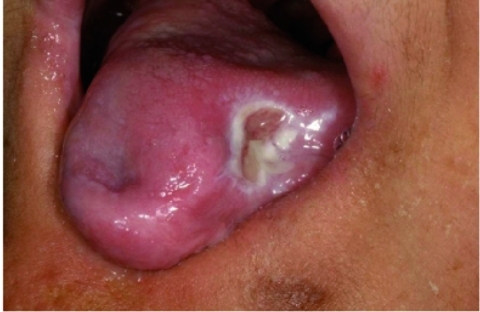
Self -mutilated tongue

**Fig. 3A and 3B:**
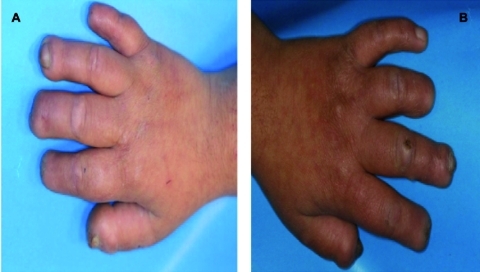
Appearance of the patient´s hands and fingers during dental treatment. Observe the foreshortening of the distal phalanges of the patient´s hands. The patient has almost no finger nails as the result of the self- mutilating behavior

In the maxilla, both the right and left permanent first molars were in good condition and only occlusal caries were noted. The other remaining maxillary teeth and roots were primary teeth with extensive caries (Figure 1A and 1B). Necrotic ulcers were apparent on the left side of the tongue and buccal mucosa which resulted from the child's biting habit (Figure 2). The patient had a decreased ability to open her mouth that was attributed to a fibrous band of scar tissue in the cheeks. The hands and fingers also showed signs of biting (Figure 3A and 3B).

**Fig. 4:**
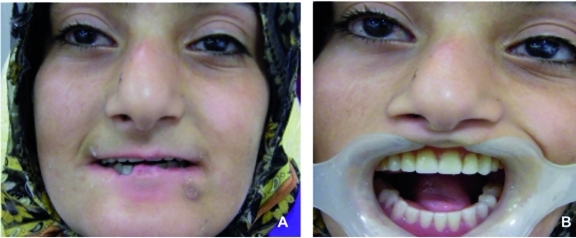
Finalization of prosthesis in the patient´s mouth

Although the patient had mild mental retardation, she was very cooperative and eager to have teeth that could improve her appearance and function. After extraction of her maxillary primary teeth and roots under anesthesia (due to the vasoconstrictor role of epinephrine), an esthetic-functional acrylic removable partial prosthesis was provided. In the mandible, the cervical caries of the canines were removed and restored with tooth-colored composite resins. After removal of the canine teeth undercuts, a mandibular overdenture was constructed (Figure 4).

The parents were instructed to remove both prostheses at night. Prophylactic daily use of fluoride gel inside the overdenture was recommended to prevent caries. During the first visit (24 hours after prosthesis insertion), the mucosa was checked carefully for any signs of tissue inflammation and ulcers due to the prosthesis. After one week, the mother reported that her daughter had managed well with artificial teeth and that she had been able to chew soft food.

At one month recall, the patient could chew tough food with her prosthesis. An improvement in ulcerations was noted and no new lesions were seen. The prosthesis improved the patient's appearance and psychosocial adjustments. After a six month follow-up, the girl was in good condition and was satisfied with her prosthesis. There was no weight loss and she could chew well. Her oral hygiene improved due to her care and the parents’ cooperation. The remaining teeth were also in good condition and no gingivitis around the canine teeth was observed.

## Discussion

Hereditary sensory and autonomic neuropathies (HSAN) are a group of disorders characterized by insensitivity to noxious stimuli and autonomic dysfunction, associated with pathological abnormalities of the peripheral nerves. Five types of HSANs have been identified by Dyck.[14] Type IV congenital insensitivity to pain with anhidrosis (CIPA) is characterized by inexplicable episodes of fever at an early age, in addition to insensitivity to pain and self-mutilation. Those affected do not sweat or cry.[15]

CIPA is secondary to a mutation in the neurotrophic tyrosine kinase receptor type I (NTRK1) gene. Mutation of this gene inhibits the development of nerve growth factor (NGF), and dependent sensory and autonomic neurons during the embryonic period. [16] NGF is not necessary for cellular survival; however, it plays a crucial role in pain generation and hyperalgia during episodes of acute and chronic pain.[17] Insensitivity to pain can cause self-mutilating behavior for these patients.[9] In most cases, bite injuries to the tongue, lips and fingers begin with the eruption of the primary teeth.[18]

Sometimes the self-mutilating behavior leads to severe injuries such as self-extraction of the teeth and nails. Oral self-mutilating behavior represents a challenge for dentists. Therefore, treatment of these patients is diverse and is predicated upon the circumstances of individual cases. In the 1960s, dentists extracted the teeth of children diagnosed with CIPA in order to avoid oral self-mutilation and full denture therapy.[7]

We believe that treatment of these patients depends on the patients’ age and intelligence, as well as the parents’ attitude and cooperation. If the patient is at an early age and the parents are cooperative, the use of a night-guard, grinding sharp edges of the teeth, or the addition of a composite are helpful; rather than the performance of a full mouth extraction which is an extremely radical treatment that causes bone loss. This radical treatment should be considered as a last alternative therapy. In older patients with mild to moderate mental retardation, routine dental work (root canal therapy), prosthodontic treatment (crowns) and/or orthodontic treatments can be considered. A rubber dam should be used in hyperkinetic patients in order to avoid serious accidents.[19] Insertion of dental implants in these patients has not been reported, but an implant placement in the ankle of a CIPA woman represents a view of future dental implant therapy in adult CIPA patients. In fact, the likelihood of chronic osteomyelitis in these patients should be considered.

Prevention of self-mutilation in CIPA patients should involve a team of multidisciplinary physicians as well as a dentist. Treatment of these patients is quite difficult and information regarding this issue is scarce in dental literature. Therefore, treatment of these patients is diverse and predicated upon individual cases. Conventional radical full mouth teeth extraction should be avoided in CIPA patients and it should be the last alternative therapy.
